# HIV-1 gp120 impairs the induction of B cell responses by TLR9-activated plasmacytoid dendritic cells

**DOI:** 10.1186/1742-4690-9-S2-P9

**Published:** 2012-09-13

**Authors:** NP Chung, K Matthews, RW Sanders, JP Moore

**Affiliations:** 1 Weill Cornell Medical College, New York, NY, USA

## Background

Plasmacytoid dendritic cells (pDCs) play a central role in innate and adaptive immunity to viral infections, including HIV-1. pDCs produce substantial quantities of type I IFN and proinflammatory cytokines upon stimulation by Toll-like receptors (TLR), specifically TLR7 or TLR9. We have studied how gp120 affects human pDC responses to TLR9 agonists, and the subsequent ability of the pDCs to stimulate B cells, with the goal of learning how better to induce B cell responses to Env protein vaccines.

## Methods

pDCs were isolated from human peripheral blood using CD304 magnetic beads, and then treated with endotoxin-free recombinant gp120 during stimulation with TLR9 agonists. IFN-α, IL-6, TNF-α, IRF-7 and BAFF were quantified at the protein or mRNA level. Co-cultures were performed to study how gp120-treatment of the pDCs affected their abilities to stimulate B cell responses, specifically proliferation, differentiation to plasma cells and IgG/IgM production.

## Results

We found that gp120 impaired IFN-α production by pDCs in response to TLR9 (CpG-ODN), but not TLR7, stimulation. Receptor-blocking studies showed the inhibitory effects were mediated via CD4 and the C-type lectin receptor BDCA-2, but not via CCR5 or CXCR4. Treatment with gp120 inhibited CpG-induced pDC maturation, TNF-α and IL-6 production and IRF-7 and BAFF mRNA expression. The gp120-treated, CpG-activated pDCs also had impaired abilities to induce B cell proliferation, plasma cell differentiation and Ig production, due at least in part to decreased expression of BAFF and other cytokines.

## Conclusion

Taken together, our data show that HIV-1 gp120 impairs pDC functions and B cell activation, and imply that TLR9 ligands may not be good adjuvants to use in combination with Env-based vaccines.

